# NMDA Receptor-Mediated Neuroprotective Effect of the *Scutellaria baicalensis* Georgi Extract on the Excitotoxic Neuronal Cell Death in Primary Rat Cortical Cell Cultures

**DOI:** 10.1155/2014/459549

**Published:** 2014-05-21

**Authors:** Jinsong Yang, Xiaohong Wu, Haogang Yu, Xinbiao Liao, Lisong Teng

**Affiliations:** ^1^Department of Radiation Oncology, The First Affiliated Hospital, College of Medicine, Zhejiang University, Hangzhou 310003, China; ^2^Department of Physical Medicine & Rehabilitation, The First Affiliated Hospital, College of Medicine, Zhejiang University, Hangzhou 310003, China; ^3^Department of Surgical Oncology, The First Affiliated Hospital, College of Medicine, Zhejiang University, Hangzhou 310003, China

## Abstract

The objective of the current research work was to evaluate the neuroprotective effect of the ethanol extract of *Scutellaria baicalensis* (S.B.) on the excitotoxic neuronal cell death in primary rat cortical cell cultures. The inhibitory effects of the extract were qualitatively and quantitatively estimated by phase-contrast microscopy and lactate dehydrogenase (LDH) assays. The extract exhibited a potent and dose-dependent inhibition of the glutamate-induced excitotoxicity in the culture media. Further, using radioligand binding assays, it was observed that the inhibitory effect of the extract was more potent and selective for the N-methyl-D-aspartate (NMDA) receptor-mediated toxicity. The S.B. ethanol extract competed with [^3^H] MDL 105,519 for the specific binding to the NMDA receptor glycine site with 50% inhibition occurring at 35.1 **μ**g/mL. Further, NMDA receptor inactivation by the S.B. ethanol extract was concluded from the decreasing binding capability of [^3^H]MK-801 in the presence of the extract. Thus, S.B. extract exhibited neuroprotection against excitotoxic cell death, and this neuroprotection was mediated through the inhibition of NMDA receptor function by interacting with the glycine binding site of the NMDA receptor. Phytochemical analysis of the bioactive extract revealed the presence of six phytochemical constituents including baicalein, baicalin, wogonin, wogonoside, scutellarin, and Oroxylin A.

## 1. Introduction

N-Methyl-D-aspartate (NMDA) receptors have a well-defined role in neuronal plasticity. Excitotoxic neuronal cell death can occur if these receptors are overactivated. As such, NMDA antagonists (NMDA inhibitors) are thought to play a crucial role in neuroprotection. However, it has been reported that NMDA receptors also have an important role in enhancing neuronal survival. Thus, it follows that NMDAR antagonists not only will protect from excitotoxicity but would also reduce prosurvival activity of NMDAR. Hence, the recognition of the switches regulating prosurvival vis-à-vis proexcitotoxic outcome of NMDAR stimulation may lead to development of NMDAR antagonists that specifically block the excitotoxicity while augmenting the protective NMDAR signaling. Glutamate is the principal excitatory neurotransmitter in the CNS. It stimulates various types of receptors including the N-methyl-D-aspartate receptors (NMDAR). NMDAR form calcium-permeable ion channels and are principal mediators of the excitotoxic cell death following excessive release of glutamate after different forms of CNS insults [1–5].

*Scutellaria baicalensis* (Labiatae family) is a plant sometimes referred to as* Huang Qin* or* Scutellariae radix* (root).* Scutellaria baicalensis* (Chinese skullcap) is a traditional Chinese medicine for the purposes of cardiovascular and cognitive health as well as longevity [6–8]. It has been reported to possess strong neuroprotective properties.* Scutellaria baicalensis* is a component of various combination therapies (from TCM) including* Ger-Gen-Chyn-Tang* [9],* Soshiho-tang* [10], and* Shuanghuanglian* [11]. Various phytochemical components have been identified in* S. baicalensis* aerial and root parts. Baicalin (baicalein-7-glucoronide) and its aglycone baicalein as well as another glycoside known as baicalein-7-O-glucoside have been reported in* S. baicalensis*. In addition, wogonoside (wogonin-7-glucuronide) and its aglycone wogonin as well as another glycoside known as wogonin-5-O-glucoside have also been reported in it. Some other phytoconstituents reported in* S. baicalensis* are Oroxylin (5,7-dihydroxy-6-methoxyflavone) and its glucoside, neobaicalein, scutellarin, and isoscutellarin; chrysin; skullcap flavone; apigenin; luteolin; 6-hydroxyluteolin, and so forth [12–16].

The phytochemicals present in* Scutellaria* species have been reported to show a range of neuroprotective effects. Wogonin inhibited inflammatory activation of microglia by reduced cytotoxicity towards cocultured PC-12 neurons, supporting an* in vitro* neuroprotective role of this flavonoid. The efficacy of wogonin was further demonstrated in two experimental brain injury models. In the 4-vessel occlusion model of transient global ischemia, wogonin decreased the death rate of hippocampal neurons, the induction of iNOS, and TNF-*β* in hippocampus, whereas, in the kainate injection model, this flavonoid markedly protected from excitotoxic brain injury. Similarly, baicalein attenuated the NO production by suppressing iNOS induction, in LPS-activated BV-2 mouse microglial cells, besides reducing apoptotic cell death and NF-kB activation [17–19].

## 2. Materials and Methods

### 2.1. Materials

The plant material (roots) of* Scutellaria baicalensis* was collected from a local region of Hangzhou and was authenticated by a well-known botanist. Minimum essential medium (MEM), horse serum, and fetal calf serum were obtained from Gibco. Multiwell plates were bought from Falcon. Laminin, poly-L-lysine, L-glutamine, Glu, glucose, NMDA, polyethylenimine, and cytosine arabinoside were purchased from Merck. [^3^H]MDL 105,519 and [^3^H]MK-801 were purchased from Amersham Biosciences, Inc., and MOLEKULA Ltd., respectively. All other chemicals were of reagent grade.

### 2.2. Preparation of the Extract

The roots of the plant were thoroughly washed with tap water, shade dried, and then chopped into small pieces. Ethanol (95%) was used for hot extraction which was carried out for 4 hours using a soxhlet extraction apparatus. The extract was then concentrated under reduced pressure in a rotary evaporator at 40°C and was then kept in a refrigerator at 4°C prior to use.

### 2.3. Primary Rat Cortical Neuronal Cultures

Primary rat cortical neuronal cultures were obtained from Sprague-Dawley (SD) rat embryos at embryonic stage of 14–16 days (Experimental Animal Centre of Sichuan University, Chengdu City, Sichuan Province, China). The rats used in the experiment weighed between 250 and 300 g. The cerebral cortices were dissected and mechanically dissociated into single cells by trituration through Pasteur pipettes. Cells were plated at a density of 6 × 10^5^ cells per well on 24-well culture plates coated with Laminin and poly-L-lysine. Then, the cell cultures were incubated at 37°C in a humidified atmosphere of 5% CO_2_ in an MEM containing medium supplemented with glucose (25 mM), fetal calf serum (5%), horse serum (5%), and glutamine (5 mM). After 14–16 days in the culture medium, the cells were used for the experiment.

### 2.4. Induction of Neuronal Cell Excitotoxicity and Their Assessment

Earle's balanced salt solution (EBSS) was used to rinse the cultured neuronal cells before the excitotoxic injuries were induced by exposure to 350 *μ*M NMDA or Glu concentration in Mg^2+^-free EBSS for 20 min. After the induction of excitotoxicity, the neuronal cultures were rinsed and maintained at 37°C for 20–24 hours in glucose supplemented MEM. Then, the neuronal cell cultures were treated for 30 min with 350 *μ*M NMDA or Glu in the presence of 1, 10, 25, 50, and 100 *μ*g/mL concentrations of the extract in order to assess the effect of the S.B. ethanol extract on NMDA or Glu-induced neuronal injury. Stock sample solutions of the ethanol extract were prepared in dimethyl sulfoxide (DMSO). Lactate dehydrogenase (LDH) assay and phase-contrast microscopy were used to estimate quantitative and qualitative extent of the neuronal damage, respectively. As described previously [20], neuronal damage quantification was estimated by measuring lactate dehydrogenase (LDH) activity released into the culture medium. Background LDH release was determined by using sister neuronal cultures in each experiment and then subtracted from the values. The final concentration of the vehicle (not more than 0.5%) exhibited no effect on cell excitotoxicity.

### 2.5. Synaptic Membranes for Receptor Binding Studies

For receptor binding studies, the synaptic membranes were prepared from forebrains of male Sprague-Dawley rats as reported earlier [21]. After centrifugation, the forebrains were homogenized at 1500 ×g for 15 min. The supernatant was collected and centrifuged at 3000 ×g for 30 min. The pellet stored at −70°C overnight was liquefied at room temperature and then again suspended in 25 mM Tris-acetate (PH 7.0) containing 0.05% Triton X-100. The solution was incubated at 37°C for 15 min and centrifuged at 4000 ×g for 15 min. Finally, the pellet was resuspended to give a protein concentration of 1.5 mg/mL, determined by DC Protein Assay Kit (Bio-Rad).

### 2.6. Binding Studies with [^3^H]MDL 105,519

[^3^H]MDL 105,519 binding assay was carried out in 96-well plates. Incubation of the synaptic membranes (25 *μ*g/well) at 25°C for 20 min in a mixture containing 4 nM [^3^H]MDL 105,519 and different concentrations of the S.B. extract along with 25 mM Tris-acetate was carried out. This reaction mixture was then washed thoroughly with the 0.6 mL of ice-cold 25 mM Tris-acetate buffer by filtration using Whatman GF/A glass fiber filter. Then, the filter was wrapped with MeltiLex, sealed in a sample bag, and then counted by MicroBeta TriLux (Microplate Scintillation and Luminescence Counter, PerkinElmer) at a counting efficiency of 30–40%. Nonspecific binding, which was determined in the presence of 1 mM glycine, was less than 10% of total binding.

### 2.7. Binding Studies with [^3^H]MK-801

For this purpose, the synaptic membranes (25 *μ*g/mL) were incubated for 40 min in a 2 mL reaction mixture containing 4 nM [^3^H]MK-801, 0.2 *μ*M Glu, 2 mM glycine, and different concentrations of the ethanol S.B. extract along with 25 mM Tris-acetate buffer (PH 7.0). Then, the reaction was terminated by filtering through Whatman GF/B glass fiber filter and the bound radioactivity was determined by a liquid scintillation counter (Hidex 300 SL, USA) at a counting efficiency of 50–55%. Nonspecific binding, determined in the presence of 200 *μ*M MK-801, was less than 10% of the total binding.

## 3. Liquid Chromatography-Tandem Mass Spectrometry (LC-ESI-MSMS)/HPLC Analysis

LC-MS equipment (LC-MS QqQ-6410B Agilent Technologies) consisted of a chromatographic system coupled with an Agilent Triple Quad mass spectrometer fitted with an ESI source. MS conditions were the following: nebulizer gas 45 Psi, gas temperature 325°C, capillary voltage 4000 V, and MS range 100–1200 Da; MSn spectra were obtained using both positive and negative modes.

HPLC analysis was carried out by an Agilent 1260 infinity series. A Chromolith RP-18e column (4.6 mm ID, 50 mm length) (Merck) was used. Gradient elution of the samples was performed using 0.1% formic acid (eluent A) and methanol (eluent B). The gradient elution initial conditions were 45% of eluent B with linear gradient to 60% from 2 to 10 min, followed by linear gradient to 70% of eluent B at 35 min, and then linear gradient to 99% of eluent B at 38 min, with this proportion being maintained for 2 min. The column was then returned to the initial condition at 40 min and maintained until the end of the run at 42 min. The flow rate was 1 mL/min. The sample injection volume was 10 *μ*L.

### 3.1. Statistical Analysis

The experiments were done in triplicate and the data were processed by nonlinear regression analysis using GraphPad software, USA, for the calculation of IC_50_ values. All the results were expressed as mean ± SEM. Statistical significance was considered at *P* ≤ 0.05.

## 4. Results

### 4.1. Assessment of Neuronal Excitotoxicity

**Figure 1:**
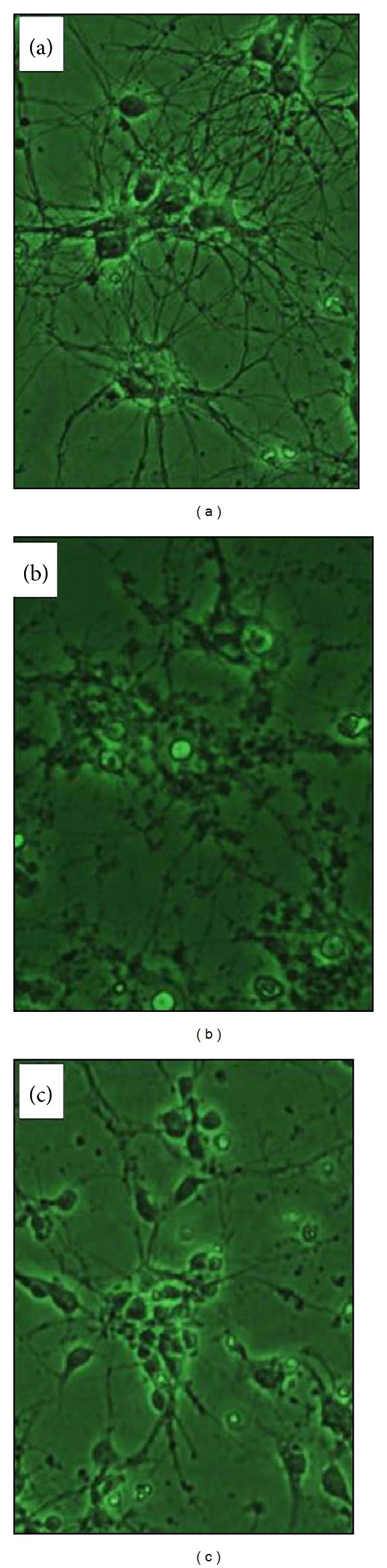
High magnification (×400) photomicrographs of cultured embryonic cortical neuronal cells from 14- to 16-day-old rats (*in vitro*); normal group (untreated). The nuclear membranes are distinct, and the dendrocyte is normal [1]. Glu-[350 *μ*M for 20 min in Mg^2+^-free EBBS and incubated for 20–24 h at 37°C in MEM supplemented with glucose] treated group, showing cellular swelling; dendrocyte is breaking and the nuclear shape is indistinct [2]. Glutamate-treated group in presence of* S. baicalensis* (100 *μ*g/mL) extract and maintained as above [3]; note that the cellular shape is generally regular almost like that of untreated group.

**Figure 2:**
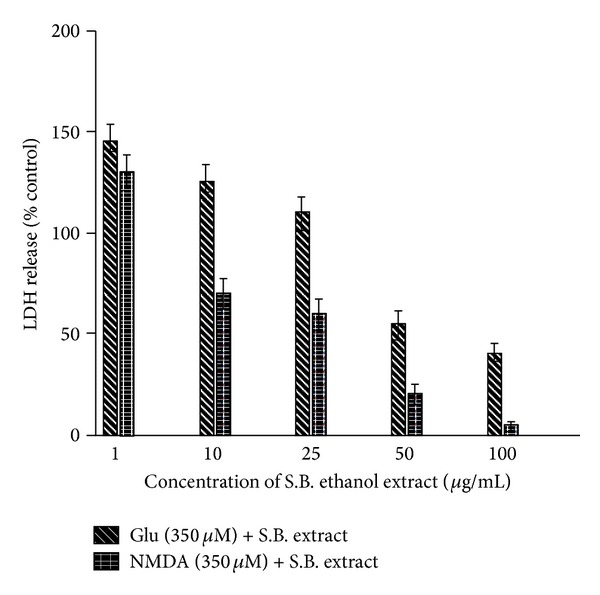
Inhibition of N-methyl-D-aspartate (NMDA) or glutamate- (Glu-) induced excitotoxicity by the ethanol extract of* Scutellaria baicalensis* (S.B.). Lactate dehydrogenase (LDH) activities given off by the damaged neurons into the culture media were measured at 20–24 h after the exposure. Data were calculated as percent of control LDH activity released into the Glu-treated culture medium. Cultures were exposed for 20 min to 350 mM Glu or NMDA in the presence of various concentrations (1, 10, 25, 50, and 100 *μ*g/mL) of the AGR extract and maintained as described in the experimental part.

After the exposure of cultured rat cortical neuronal cultures to 35 *μ*M Glu or NMDA for 20 min, acute neuronal swelling, breakage in dendrocytes, and the indistinct nuclear shape were observed by phase-contrast microscopy (Figure 1). This neuronal injury became more intense with increase in incubation time, ultimately resulting in enhanced neuronal damage and finally neuronal cell death. The objective of the current research work was to evaluate the neuroprotective effect of the ethanol extract of* S. baicalensis* as claimed in the traditional Chinese medicine, where this plant has been used against various neurological disorders. Our results demonstrated that when the neuronal cell cultures were exposed for 20 min to Glu (350 *μ*M) in the presence of different concentrations of the S.B. extract, the Glu-induced excitotoxicity was dramatically inhibited in a dose-dependent pattern showing maximum inhibition at 100 *μ*g/mL of the extract (Figure 1). The qualitative estimation of Glu-induced neuronal cell death was evaluated by phase-contrast microscopy, whereas the degree of neuronal cell death was quantitatively estimated by calculating LDH release activities given off by the damaged neurons out into the culture media. The IC_50_ values of the extract were found to be 60.01 and 28.60 *μ*g/mL, respectively, for Glu and NMDA-induced excitotoxicity, respectively (Figures 1 and 2).

### 4.2. Selective Inhibition of NMDA Receptor-Mediated Excitotoxicity

As the Glu-induced excitotoxicity is arbitrated through a number of Glu-receptor subtypes, in order to estimate whether the S.B. extract inhibits the NMDA receptor-mediated toxicity, the cell cultures were subjected to 350 *μ*M NMDA for 20 min. NMDA-induced excitotoxicity is already reported in the literature [22]. In the current study, we found that neuronal damage induced by NMDA and the neuronal deformation was morphologically similar to that produced by Glu. The S.B. extract exhibited a potent inhibition of NMDA-induced excitotoxicity as well. As is evident from the graph (Figure 2), the NMDA-inhibition curve exhibited a more pronounced shift than Glu-inhibition curve, indicating a more powerful and selective inhibitory effect of the extract on NMDA receptor-mediated excitotoxicity. The IC_50_ value of the extract in this case was found to be 28.6 *μ*g/mL. The extract showed a dose-dependent inhibition, and at a concentration of 100 *μ*g/mL, almost 90–95% of the neurons were secured from the excitotoxic insults. As can be seen in Figure 1, after the treatment with 100 *μ*g/mL S.B. extract + 350 *μ*M Glu, the neurons showed regular and distinct shape under phase-contrast microscopy unlike in 350 *μ*M Glu culture. Thus, these results indicate that the ethanol S.B. extract displays a neuroprotective effect against Glu or NMDA-induced excitotoxic neuronal cell death and more importantly this neuroprotective effect is principally arbitrated through NMDA receptors.

### 4.3. [^3^H]MDL 105,519 Binding Studies

**Figure 3:**
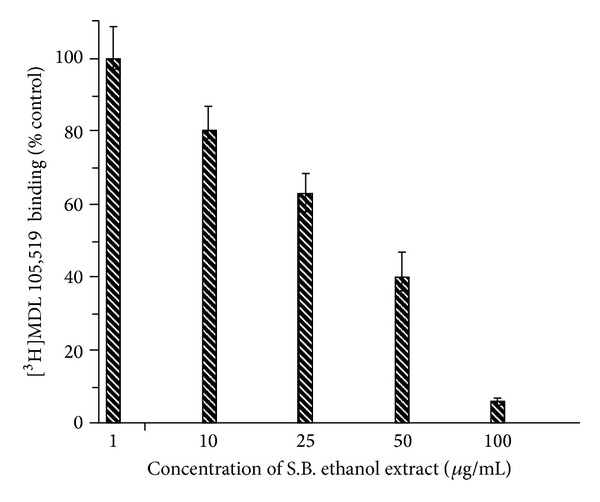
*Scutellaria baicalensis* (S.B.) extract inhibits the specific binding of [^3^H]MDL 105,519 to the glycine site of the N-methyl-D-aspartate (NMDA) receptor. Different concentrations of the extract were employed. Nonspecific binding determined in the presence of 2 mM glycine was subtracted from the total binding. Data were calculated as percent of the control binding measured in the absence of the extract.

In the present study, further experiments were carried out to confirm whether the S.B. extract interacts with the glycine site of NMDA receptor. For this purpose, [^3^H]MDL 105,519 radioligand receptor binding assay was carried out where [^3^H]MDL 105,519 acts as a selective glycine site antagonist. The results indicated that S.B. extract competitively inhibited the binding of [^3^H]MDL 105,519 to the glycine receptor site (Figure 3). It was observed that, at a concentration of 100 *μ*g/mL of the extract, more than 90% of the binding of [^3^H]MDL 105,519 was displaced by the extract. The IC_50_ value was found to be 35.1 *μ*g/mL.

### 4.4. [^3^H]MK-801 Binding Studies

**Figure 4:**
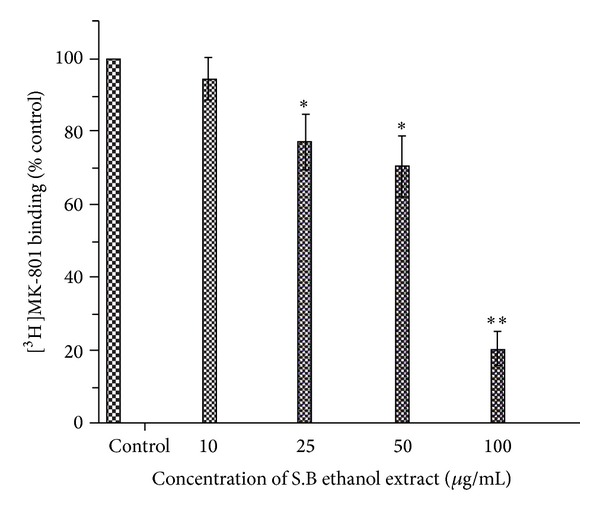
The ethanol extract of* Scutellaria baicalensis* inhibits the binding of [^3^H]MK-801 to the N-methyl-D-aspartate (NMDA) receptor. Different concentrations of the extract (here we used 10, 25, 50, and 100 *μ*g/mL) were employed. Nonspecific binding determined in the presence of 200 *μ*M MK-801 was subtracted from the total binding. Data were calculated as percent of the control binding measured in the absence of the extract. Each point represents the mean ± S.D. *P < 0.05; **P < 0.01 (compared with control binding).

As can be seen in Figure 4, the specific binding of [^3^H]MK-801 was appreciably inhibited by the S.B. extract. The IC_50_ value of the extract was found to be 65.1 *μ*g/mL. At a concentration of 100 *μ*g/mL, [^3^H]MK-801 binding with the receptor site was around 20% of the control binding calculated in the absence of the extract. Based on these two radioligand binding assays, we can conclude that the S.B. extract probably inhibits the NMDA receptor by knocking out glycine from its binding site. It has been already reported in the literature that assessment of [^3^H]MK-801 binding is a hallmark of the functional state of NMDA receptors [23].

### 4.5. LC-ESI-MSMS Analysis/HPLC Analysis

**Figure 5:**
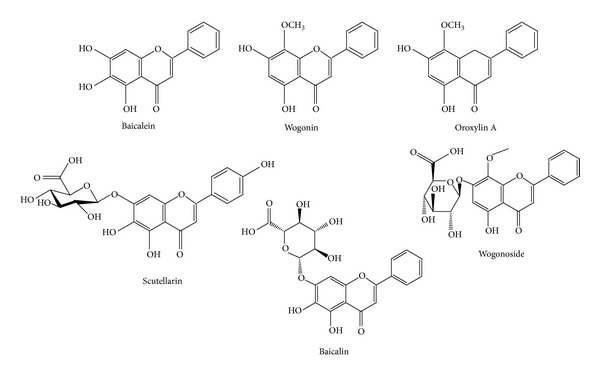
Phytochemical compounds identified in the bioactive ethanol extract of the root of* Scutellaria baicalensis* Georgi. These compounds were identified using LC-ESI-MS analysis.

**Figure 6:**
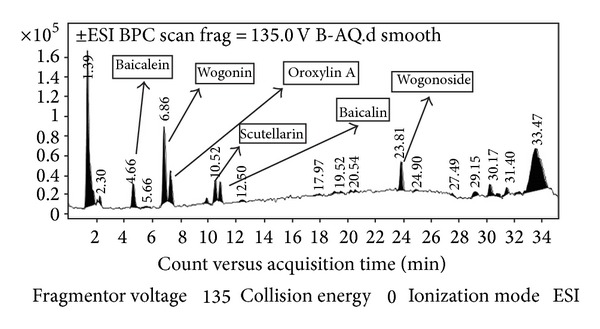
LC-MS chromatogram of the bioactive ethanol extract of the root of* Scutellaria baicalensis* Georgi.

**Figure 7:**
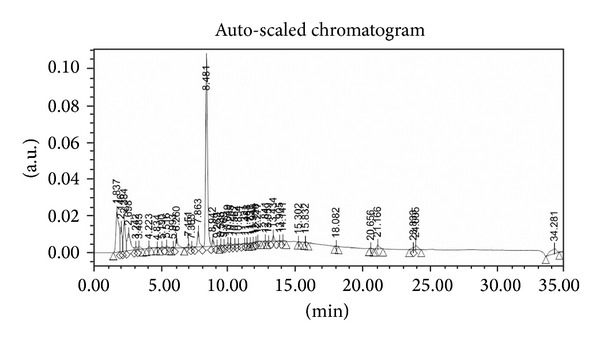
HPLC chromatogram of the ethanol extract of* Scutellaria baicalensis*.

**Figure 8:**
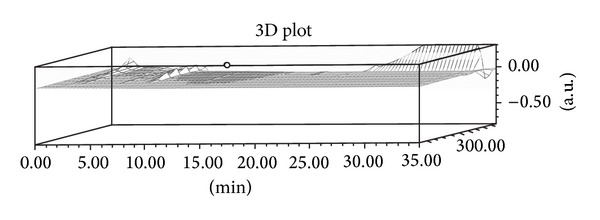
HPLC-3D plot of the ethanol extract of* Scutellaria baicalensis.*

The phytochemical analysis of the* S. baicalensis* ethanol extract was carried out by LC-ESI-MS in combination with HPLC-DAD analytical techniques. The extract was run under both positive and negative ESI-MS conditions and it showed several major and minor ionic fragments. The six chemical constituents identified were baicalein, baicalin, wogonin, wogonoside, scutellarin, and Oroxylin A (Figure 5). These phytochemicals present in the root part of* S. baicalensis* have already been reported previously in this and other plant species of the* Scutellaria* genus. The total ion MS chromatogram (TIC), HPLC profile, and HPLC-3D plot are shown in Figures 6, 7, and 8, respectively. Fragmentation of the major peaks was used for the identification of compounds. The identification of the chemical compounds was also carried out by comparing the molecular ion peaks along with the MS fragmentation pattern with those of the literature.

## 5. Discussion

*Scutellaria baicalensis* Georgi has been reported to contain various flavonoids such as baicalein (5,6,7-trihydroxyflavone), baicalin (baicalein-7-O-glucuronide), and wogonin (5,7-dihydroxy-8-methoxyflavone). These phytochemicals are proven potent antioxidants and have been reported to quench reactive oxygen species (ROS) to protect neuronal cells from oxidative damage in cerebral ischemia/reperfusion. These flavonoids have also been reported to inhibit lipid peroxidation of neuronal membranes and to prevent Glu-induced excitotoxicity. Wogonin, as an inhibitor of the CNS inflammation, has been reported to suppress NO (nitric oxide) production and iNOS (inducible nitric oxide synthase) activation in cultured rat astrocytes. Baicalin, another phytochemical from* S. baicalensis,* has been reported to enhance neural stem/progenitor cell production and hippocampal dependent neurogenesis in rats following cerebral ischemia when these rats were injected with 50 mg/Kg daily [24]. Baicalin has also been reported to preserve heat shock protein 70 (HSP70), with the latter being known to exert protective effects in neurons [25–27].* S. baicalensis* as such has been shown to exhibit memory enhancing properties against ibotenic acid (toxin), chronic lipopolysaccharide infusion, gamma-irradiation, beta amyloid proteins, ischemia, and so forth [28–31]. Ethanol extract of* Scutellaria baicalensis* Georgi prevents oxidative damage and neuroinflammation and memorial impairments in artificial senescence mice [32]. The amount of baicalin in* Scutellaria* is much more than that of baicalein. After oral administration of the aqueous extract of* S. baicalensis*, baicalein, baicalin, and wogonin are rapidly absorbed. Baicalin is metabolized into baicalein by bacteria prior to intestinal absorption, with the latter metabolite being detected in plasma up to 24 h. In rat, baicalein enters brain crossing blood brain barrier and distributes in cortex, hippocampus, striatum, thalamus, and brain stem in 20 minutes [33, 34].

During an ischemic stroke or status epilepticus, brain glutamatergic receptors of the N-methyl-D-aspartate (NMDA) subtype are overactivated. This results in a substantial intracellular calcium rise which triggers a lethal cascade of events leading to neuronal death. NMDA receptor overactivation is also supposed to be implicated in a range of neurodegenerative diseases such as Alzheimer's disease, Huntington's chorea, and AIDS dementia syndrome. In the hunt for neuroprotective agents, NMDA receptor antagonists undoubtedly remain potential therapeutic drugs. In the present study, we identified one such NMDA receptor antagonist (*Scutellaria baicalensis* ethanol extract) which exhibits neuroprotective effect, and more importantly this neuroprotective action was mainly mediated through NMDA receptors [35].

## 6. Conclusion

In conclusion, our study convincingly demonstrated that the neuroprotective effect of the S.B. ethanol extract on the Glu or NMDA induced excitotoxicity was mediated through the blockade of NMDA receptors. So this extract can act as NMDA receptor antagonist and these may find potential applications for the discovery of neuroprotective agents from plant sources.
